# Supplementary figures and images for: Sequential early-life viral infections modulate the microbiota and adaptive immune responses to systemic and mucosal vaccination
Source: PLoS Pathog. 2024 Oct 2;20(10):e1012557. doi: 10.1371/journal.ppat.1012557 (PMC11472911; doi:10.1371/journal.ppat.1012557)

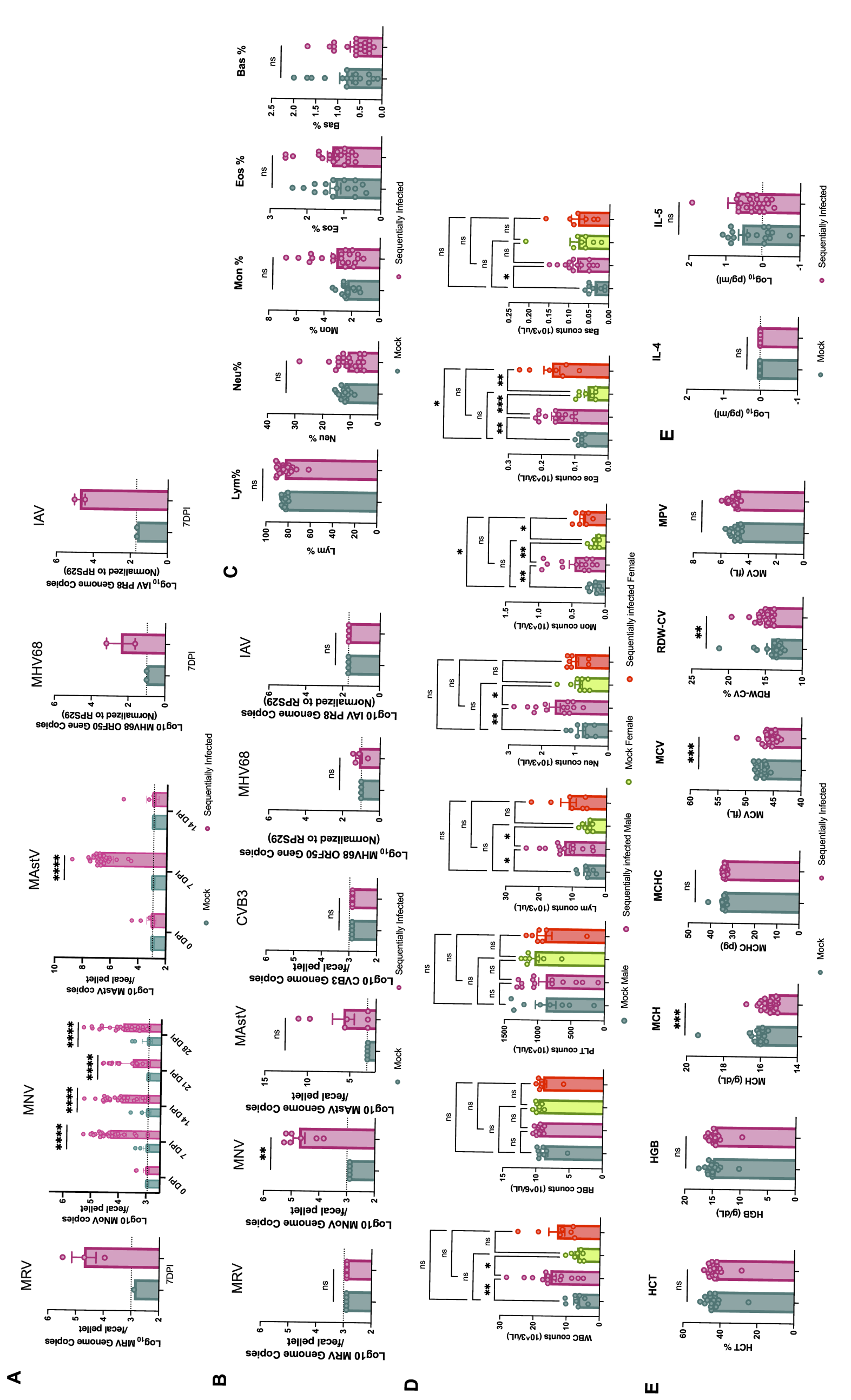

Supplement: S1 Fig — (A) Murine rotavirus (MRV) genome copies detected in fecal pellets of naïve (n = 1) and sequentially infected (n = 3) mice at 7 days post-inoculation. Murine norovirus strain CR6 (MNV) and murine astrovirus (MAstV) genome copies detected in fecal pellets of mock- (n = 18) and sequentially-infected (n = 25) mice at indicated timepoints post-inoculation. Murine gammaherpesvirus (MHV68) ORF50 gene copies were detected in the spleen in naïve (n = 2) and sequentially infected (n = 2) mice at 7 days post-inoculation. Influenza virus strain PR8 (IAV) genome copies were detected in lung tissue in naïve (n = 2) and sequentially infected (n = 2) mice at 7 days post-inoculation. Columns show the mean values, error bars depict standard deviation, and the dotted lines depict the LOD for the assays. Undetectable samples were given a value of LOD. (B) MRV, MNV, MAstV, and CVB3 genome copies were detected in fecal pellets of naïve (n = 5) and sequentially infected (n = 7) mice at 10 weeks of age. MHV68 ORF50 gene copies were detected in the spleen of naïve (n = 4) and sequentially infected (n = 4) mice at 10 weeks of age. IAV genome copies were detected in lung tissue in naïve (n = 4) and sequentially infected (n = 4) mice at 10 weeks of age. Columns show the mean values, error bars depict standard deviation, and the dotted lines depict the LOD for the assays. Undetectable samples were given a value of LOD. (C) Frequency of lymphocytes (Lym), neutrophils (Neu), monocytes (Mon), eosinophils (Eos), and basophils (Bas) in the hematological analysis of mock- (n = 16) and sequentially-infected (n = 21) mice at 10 weeks of age. (D) Separation of CBC data by sex for mock- and sequentially-infected mice. (E) Hematological analysis of hematocrit (Hct), hemoglobin (Hgb), mean corpuscular hemoglobin (MCH), mean corpuscular hemoglobin concentration (MCHC), mean corpuscular volume (MCV), mean platelet volume (MPV) and increased red cell distribution width (RDW-CV). (F) Serum cytokines IL-4 and [file ppat.1012557.s001.tif]

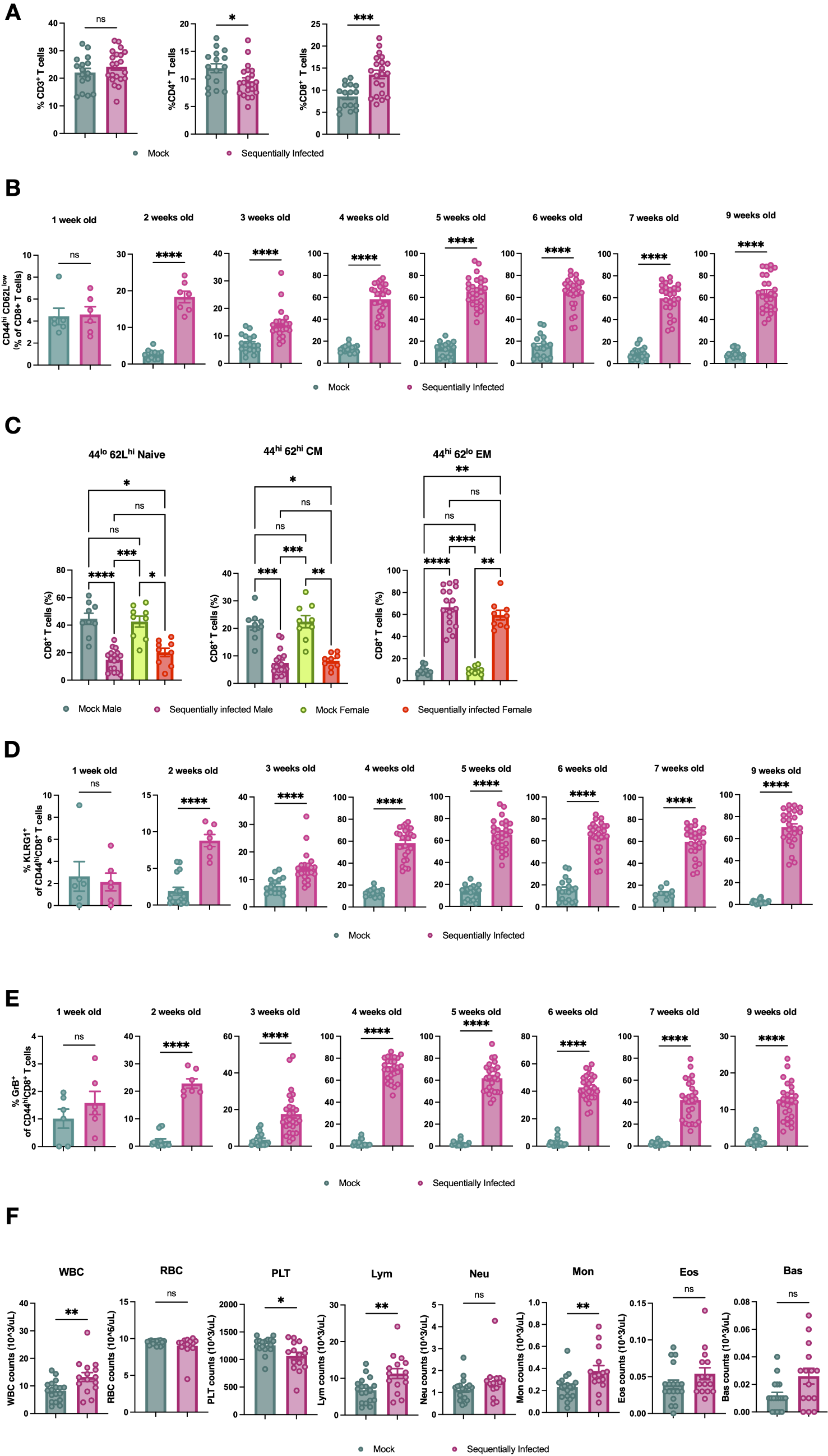

Supplement: S2 Fig — (A) Frequency of PBMC CD3+, CD4+, and CD8+ T cells from mock- (n = 16) or sequentially-infected (n = 21) mice at 10 weeks of age. (B) Frequency of CD44hi (antigen-experienced) CD8+ T cells from PBMCs of mock- or sequentially-infected mice between 1 week and 9 weeks of age. (C) CD44lo/CD62Lhi (Naive), CD44hi/CD62Lhi (antigen-experienced central memory, CM), and CD44hi/CD62Llo (antigen-experienced effector memory, EM) CD8+ T cells of mock-infected and sequentially infected mice, separated by sex. (D) Frequency of KLRG+ CD44hi CD8+ T cells from PBMCs of mock- or sequentially-infected mice between 1 and 9 weeks of age. (E) Frequency of GrzB+ CD44hi CD8+ T cells from PBMCs of mock- or sequentially-infected mice between 1 and 9 weeks of age. (F) Absolute cell counts of white blood cells (WBC), red blood cells (RBC), platelets (PLT), lymphocytes (Lym), neutrophils (Neu), monocytes (Mon), eosinophils (EOS), and basophils (BAS) in the hematological analysis of mock- (n = 14) and sequentially-infected (n = 15) mice at 7 months of age. Columns show median values, error bars represent the standard error of the mean. In A, significance was determined using unpaired Mann-Whitney test; In B, D E and F, significance was determined using a two-way ANOVA test with the Geisser_Greenhouse correction; In C, significance was determined using Dunn’s multiple comparisons correct test: *p < 0.05; **p < 0.01; ***p < 0.001; ****p < 0.0001; ns, not significant. (TIF) [file ppat.1012557.s002.tif]

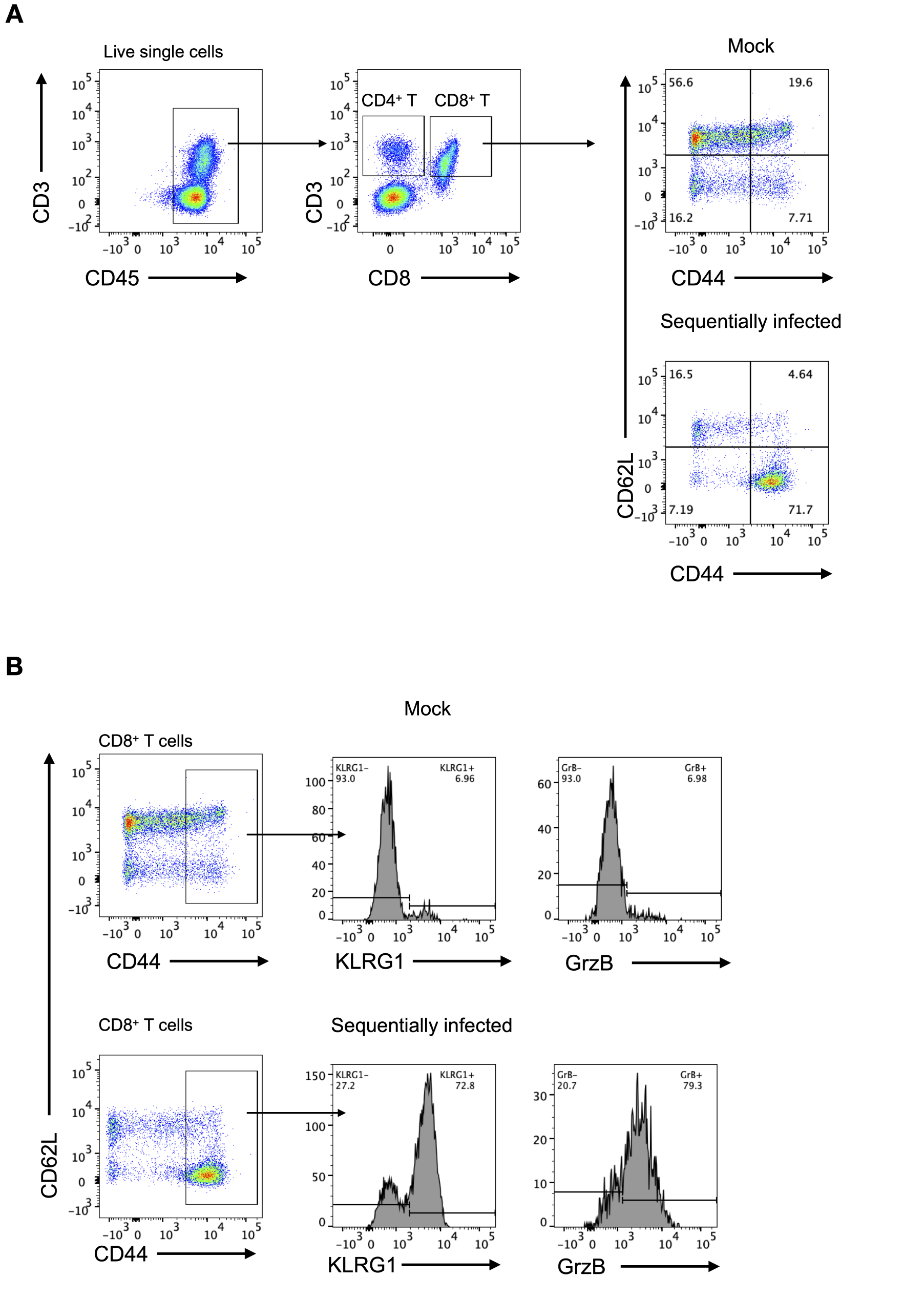

Supplement: S3 Fig — (A) T cells from mock- (n = 16) or sequentially-infected (n = 21) mice were analyzed at 10 weeks of age. PBMC live single cells were gated for CD45+ followed by CD3+CD4+ or CD3+CD8+ cell populations. CD44lo/CD62Lhi (Naive), CD44hi/CD62Lhi (antigen-experienced central memory, CM), and CD44hi/CD62Llo (antigen-experienced effector memory, EM) CD8+ T cells were evaluated with CD44 and CD62L expression. (B) Gating of KLRG1+ and GrzB+ cells from CD44hi (antigen-experienced) CD8+ T cells from PBMCs of mock- or sequentially-infected mice. (TIF) [file ppat.1012557.s003.tif]

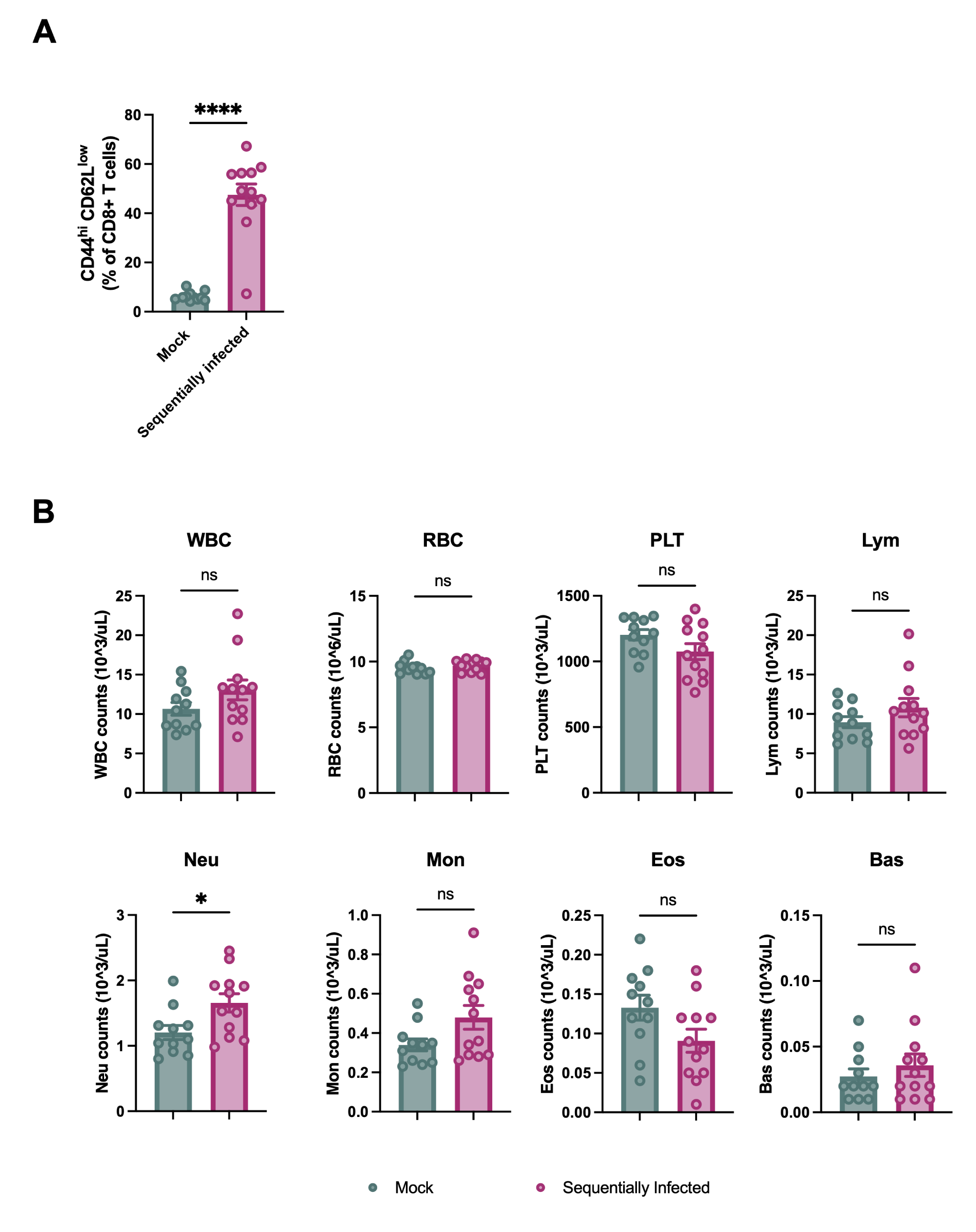

Supplement: S4 Fig — (A) Frequency of CD44hi/CD62Llo (antigen-experienced effector memory, EM) CD8+ T cell between mock-(n = 11) and sequentially-infected (n = 12) adult mice at 10 weeks post first infection. (B) Absolute cell counts of white blood cells (WBC), red blood cells (RBC), platelets (PLT), lymphocytes (Lym), neutrophils (Neu), monocytes (Mon), eosinophils (EOS), and basophils (BAS) in the hematological analysis of mock- (n = 11) and sequentially-infected (n = 12) adult mice at 10 weeks post first infection. Columns show median values, error bars represent the standard error of the mean. Significance was determined using unpaired Mann-Whitney test: *p < 0.05; **p < 0.01; ***p < 0.001; ****p < 0.0001; ns, not significant. (TIF) [file ppat.1012557.s004.tif]

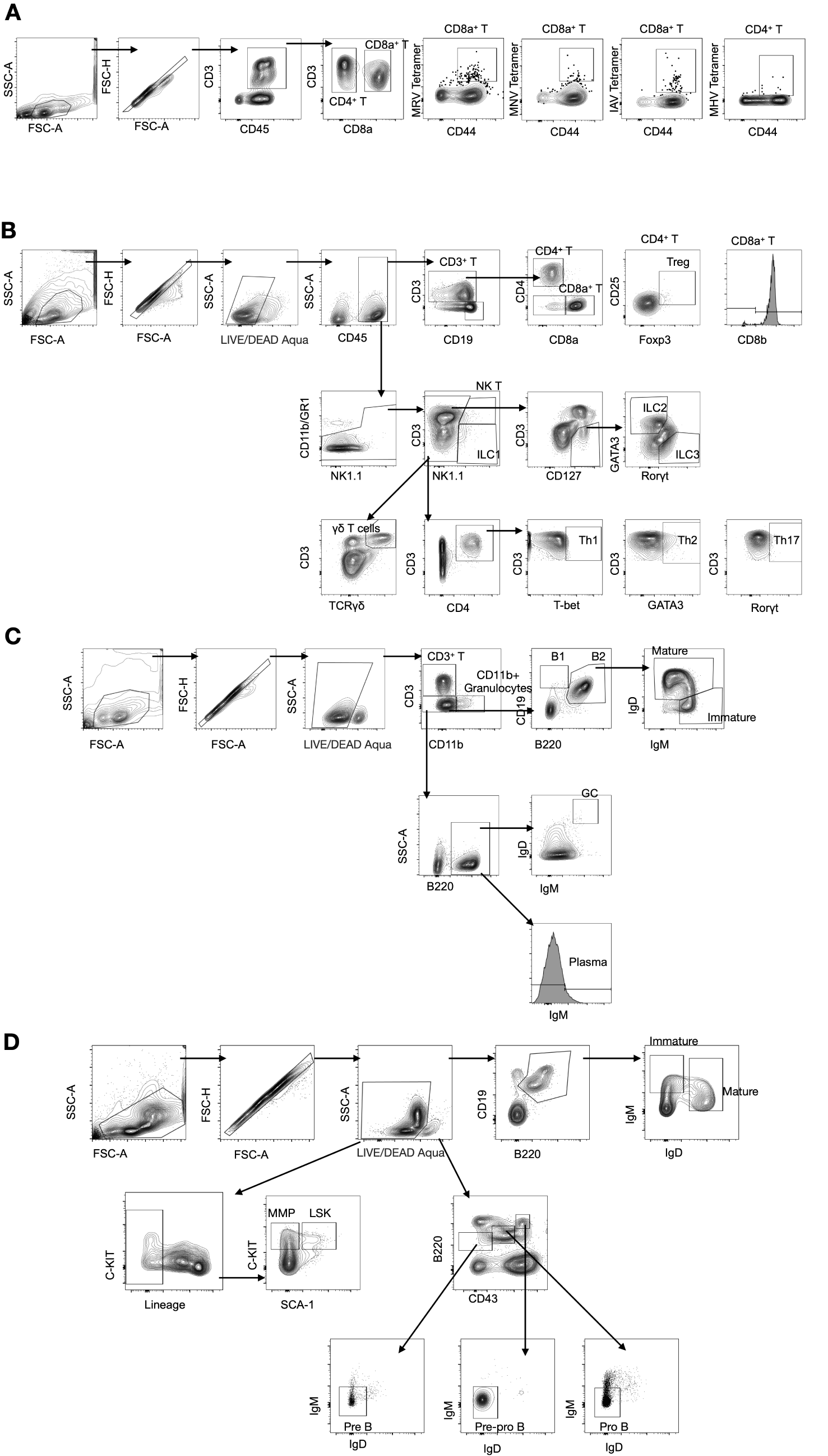

Supplement: S5 Fig — Ten-week-old mock- and sequentially-infected mice were used to analyze immune cell profiles in different tissues. (A) Splenocytes were gated for lymphocytes (FSC-A/SSC-A), singlets (SSC-W/SSC-H), and T cells (CD3/CD45) followed by CD44+ and tetramer+ cell populations to identify viral-specific T cells. (B) Lung cells were gated for lymphocytes (FSC-A/SSC-A), singlets (SSC-W/SSC-H), live cells (Aqua-), CD45+ followed by CD3+ or NK1.1+ cell populations to identify ILC, innate lymphoid cells; Th, CD4 T helper cells; Treg, regulatory CD4 T cells; γδ T, gamma delta T cells. Single positive staining for T-bet, Gata3, and Rorγt were used to define Th1, Th2, and Th17 lineages, respectively. (B) Gating of CD3+ T cells, CD11b+ granulocytes, B1, mature or immature B cells, germinal center (GC) B cells and plasma cells from splenocytes of mock- or sequentially-infected mice. (C) Gating of Lin-Sca1+c-Kit+ (LSK), mature or immature B cells, Pre B cells, Pro B cells, and Pre- Pro B cells from bone marrow of mock- or sequentially-infected mice. (TIF) [file ppat.1012557.s005.tif]

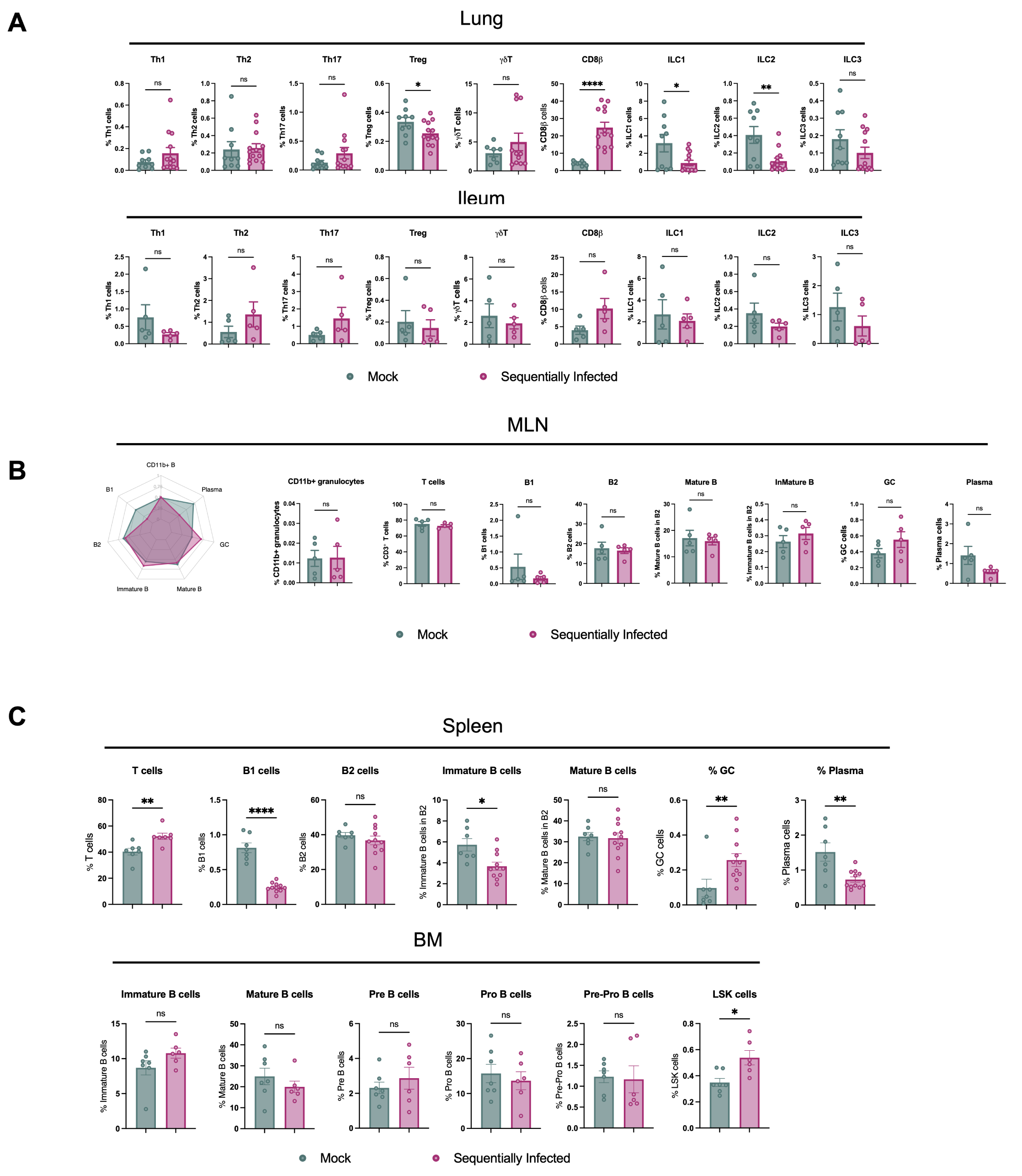

Supplement: S6 Fig — (A) Frequency of ILC, innate lymphoid cells; Th, CD4 T helper cells; Treg, regulatory CD4 T cells; γδ T, gamma delta T cell in the lung and ileum of mock- and sequentially-infected mice at 10 weeks of age. (B) Immune cell types isolated and enumerated by flow cytometry from the mesenteric lymph node (MLN) of mock- and sequentially-infected mice, including granulocytes, T cells, B1 cells, B2 cells, mature B cells, immature B cells, GC and plasma cells. (C) Frequency of T cells, B1 cells, B2 cells, mature B cells, immature B cells, GC and plasma cells from the spleen as well as the mature B cells, immature B cells, Pre B cells, Pro B cells, Pre-pro B cells, and LSK cells from bone marrow (BM) of mock- and sequentially-infected mice at 10 weeks of age. Columns show median values, error bars represent the standard error of the mean. Significance was determined using unpaired Mann-Whitney test: *p < 0.05; **p < 0.01; ***p < 0.001; ****p < 0.0001; ns, not significant. (TIF) [file ppat.1012557.s006.tif]

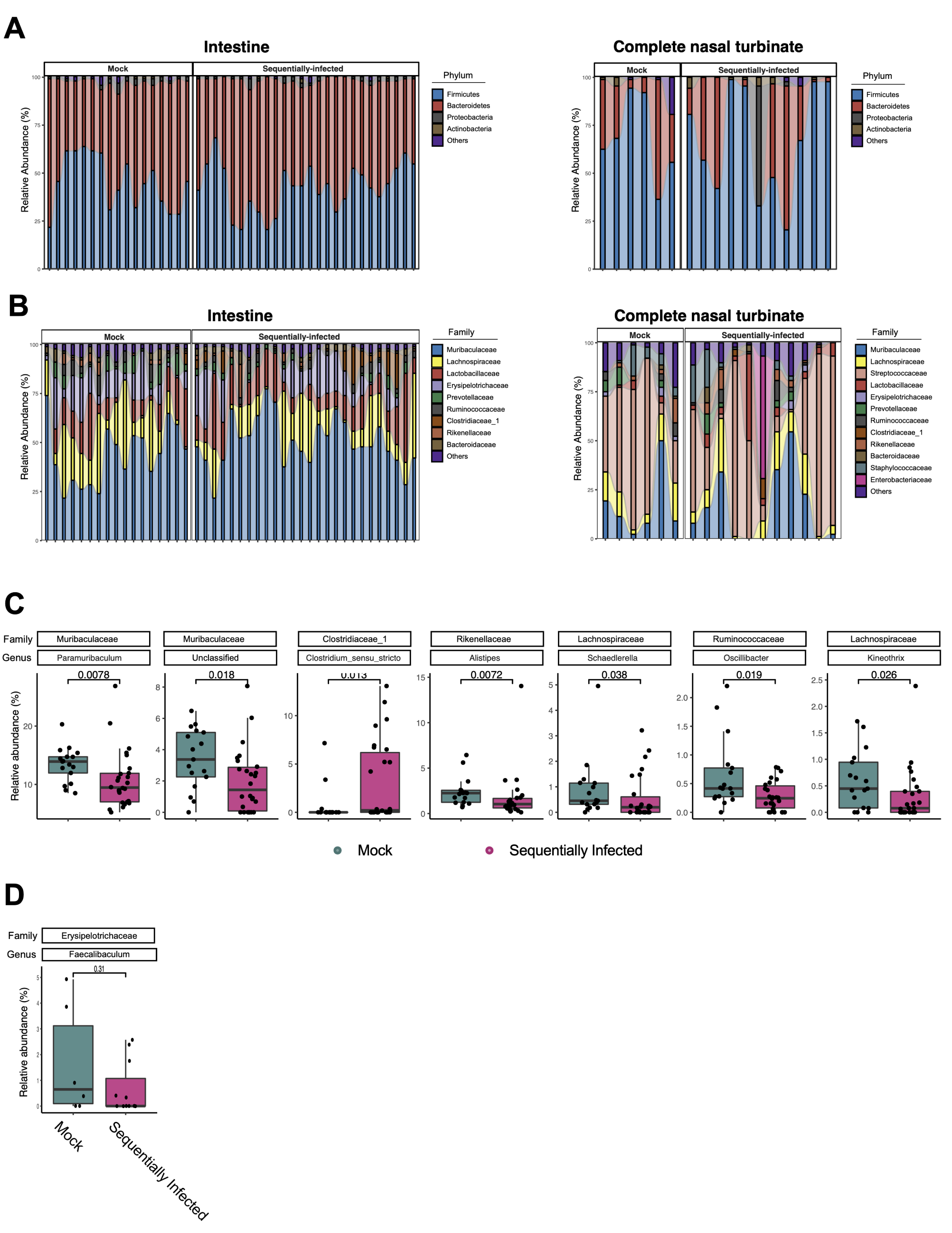

Supplement: S7 Fig — (A, B) Intestine (mock-: n = 17, sequentially-infected: n = 26) and complete nasal turbinate (mock-: n = 6, sequentially-infected: n = 11) microbiota composition in mock- and sequentially-infected mice at (A) phylum and (B) species levels. (C, D) Relative abundance of potential discriminant taxa from linear discriminant analysis within the (C) intestinal and (D) complete nasal turbinate at the genus level. (TIF) [file ppat.1012557.s007.tif]

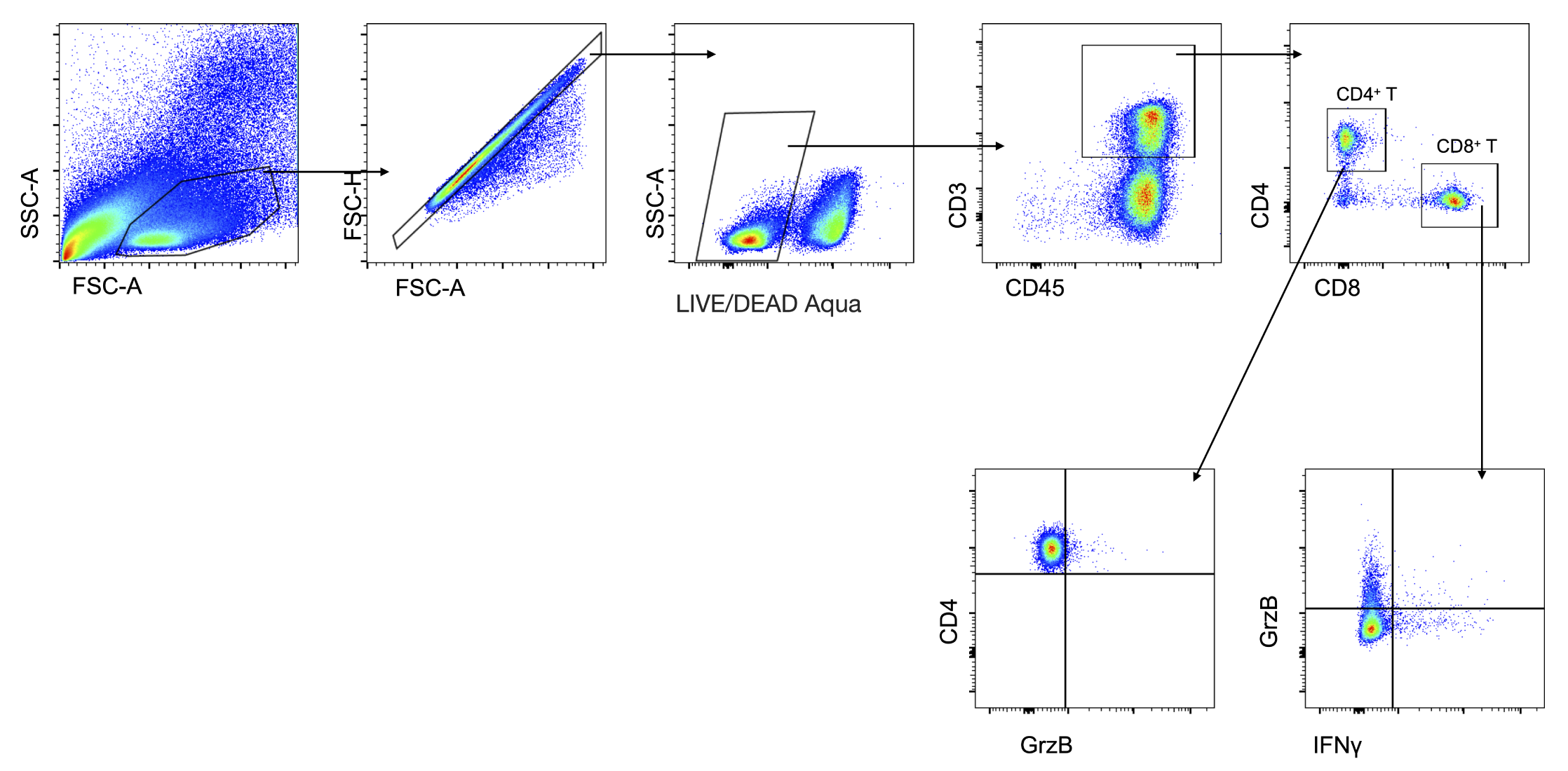

Supplement: S8 Fig — Ten-week-old mock- and sequentially-infected mice were immunized with ChAd-SARS-CoV-2-S. T cell responses were analyzed in splenocytes at 5 weeks post-vaccination. Cells were gated for lymphocytes (FSC-A/SSC-A), singlets (SSC-W/SSC-H), live cells (Aqua-), CD45+, CD19- followed by CD4+ or CD8+ cell populations expressing IFNγ or granzyme B. (TIF) [file ppat.1012557.s008.tif]
